# Consideration of Future Consequences Affects the Perception and Interpretation of Self-Conscious Emotions

**DOI:** 10.3390/bs13080640

**Published:** 2023-08-01

**Authors:** Hyeman Choi

**Affiliations:** Department of Psychology, Gachon University, Seongnam 13120, Republic of Korea; choih@gachon.ac.kr; Tel.: +82-31-750-2663

**Keywords:** emotion processing, consideration of future consequences, shame, guilt, future change, social cognition, self-conscious emotion

## Abstract

Differentiating guilt from shame expressed by others is important because self-conscious emotions have disparate behavioral consequences. The present study examined whether a future-relevant nature of an event (i.e., future opportunity) and an observer (i.e., consideration of future consequences) can impact the perception and interpretation of shame and guilt experiences. Participants (*N* = 109) read a scenario that described a target person who neglected his duty on a team project, then rated their perceived intensity of the target’s shame and guilt and their estimation of future behavior change by the target. The results showed that the participants who cared about distant future consequences (i.e., high in consideration of future consequences) thought the target person would change in the future when they believed that the target person would have an opportunity (vs. no future opportunity) to change the outcome of the event. This effect was fully mediated by the guilt intensity ratings, indicating that guilt signals future behavior change. The implications for the value of communicating self-conscious emotions are discussed.

## 1. Introduction

Although shame and guilt fall under the category of self-conscious emotions, they are distinct in various aspects (see [1,2] for a review). For example, guilt arises when people evaluate their wrong behavior, whereas shame arises when they evaluate their entire self negatively [3]. In particular, previous research has shown that individuals who experience guilt (vs. shame) have a greater potential to change their behavior in the future (e.g., [4]. Would such differences in the experience of self-conscious emotions hold true when expressed and communicated? Considering that each emotion has distinct social functions [5], shame and guilt should carry different social information (e.g., likelihood of future change in behaviors). However, little is known about the difference in social meanings that shame and guilt expressions convey to social observers. Thus, the present study attempts to fill the gap in the literature by examining how social others perceive and interpret expressions of shame and guilt. This line of research is important because communication of self-conscious emotions can affect moral judgment [6], legal decision making [7], and one’s social effectiveness [8].

However, research has shown that there is no difference in people’s expectations for a target’s future behavioral change when the target expresses shame versus guilt [9]. Recently, Choi [10] suggested that social observers need additional contextual information to distinguish any subtle differences in the social meanings of shame and guilt. Participants in Choi [10] judged a target person to be more hypocritical when the target publicly expressed guilt (vs. shame) after making an immoral decision, and then committed the same act again. Such difference in hypocrisy ratings reflects different levels of expectation of behavioral change, as hypocrisy judgment involves inconsistency detection. However, it should be noted that the participants in the shame and guilt conditions did not differ in their ratings of a direct measure of changed expectations. It seems that further context that captures the difference in future behavior change is needed for social observers to clearly understand the social meanings that shame and guilt signal. Therefore, the present study directly manipulated the future relevant aspects of an event and an observer.

The present study examined whether the perception of guilt and shame can be influenced by the degree of future opportunity for the target person to change the outcome of a past event. It was hypothesized that people would view the target person as experiencing more intense guilt, but not shame, for what they have done (e.g., cheating on tax work) when people believed that there would be a high future opportunity for the target person to be in a similar situation again (e.g., a taxpayer reports tax annually). The rationale is that, if guilt has greater implications for future behavior than shame does, then people’s perceptions of others’ guilt (vs. shame) feelings would be influenced to a greater degree by the future opportunity status of an event.

There are both cognitive and motivational reasons for seeing different levels of guilt that are proportional to the degree of perceived future opportunities. First, when there is a future opportunity to interact with a target person, people seem to experience greater guilt, but not shame, because it helps re-establish the relationship by motivating the experiencer to invest more effort in the broken relationship. In fact, people experience guilt predominantly in close relationships [11], such as with family members or friends, with whom a future interaction is highly likely. Relatedly, people experience more intense regret when a future opportunity is open (vs. closed) because it facilitates future improvement [12,13]. Given that people infer others’ emotional experiences by extrapolating their own experiences [14], people would expect that the target person would feel more intense guilt when there is a future opportunity (vs. no opportunity) for the target person to change the outcome of an event.

Second, people are motivated to prevent a negative event from happening again, because a negative event is an aversive experience. Thus, people would be motivated to see guilt—that promises future change—in a perpetrator because it alleviates the perceiver’s concern for the immoral act being committed again in the future. Indeed, people want to see redemptive, not corruptive life stories [15]. Importantly, research has shown that people’s motivation can influence their perceptions of others’ emotional states in a way that helps fulfill the perceiver’s ongoing motivation (e.g., [16]). This implies that people’s motivation to see more intense guilt can make the perceiver actually see more intense guilt. In essence, people see what they want to see [17,18].

In light of the preceding analysis regarding one’s emotional experience and motivated perceptions about others’ emotions under different levels of future opportunities, an individual difference in sensitivity to future opportunities is a particularly important variable to consider. That is, some individuals only care about the immediate consequences and not the distant future consequences of their present behaviors and decisions, whereas others care greatly about the distant future consequences of their current behavior [19]. Research on health behaviors has shown that people with high consideration of future consequences (CFC) are better at regulating themselves, for instance, in regulating healthy eating [20] and protecting themselves from COVID-19 [21]. Additionally, people with high (vs. low) CFC are more motivated by their future images (i.e., ideal possible selves) than prototypes (present selves), indicating that people with high (vs. low) CFC respond more to future consequences [22]. Thus, it appears that people with high (vs. low) CFC are more sensitive to whether or not the event provides a future opportunity to fix the problem. Indeed, Cohen, Panter, Turan, Morse, and Kim [23] reported that CFC is positively related with guilt-prone traits and negatively related with shame-prone traits, suggesting that people with high (vs. low) CFC are more prone to guilt, but not shame, experiences.

Connecting the findings of Cohen et al. [23] with the aforementioned cognitive and motivational account for our hypothesis, it was expected that people with high CFC, who are sensitive to the presence and absence of future opportunities, would see more intense guilt on others’ faces when future opportunities for the target person were still open compared to when they were closed. Arguably, if guilt carries implications for future change, seeing others experiencing guilt is important for people who care about future consequences, especially when the event has a high chance of occurring again. However, people with low CFC would not differ in their ratings of guilt intensity based on the level of future opportunity. As for the shame intensity rating, it was expected that there would be no difference in future change ratings as a function of future opportunity because shame bears less, if any, meaning regarding future change.

## 2. Methods

### 2.1. Participants and Design

One hundred and nine undergraduates from a large mid-west university participated for research credit (*M*_age_ = 19.48, *SD* = 1.44; 72.5% female; 74.3% Caucasian, 15.6% Asian/Asian American, 5.5% African American, 0.4% Hispanic/Latino, 3.7% other). The participants were randomly assigned to either high (*n* = 55) or low (*n* = 54) future-opportunity conditions. To measure individual differences in considerations of future consequences (CFC), all the participants were asked to complete the CFC scale [19] at the end of the study. A sensitivity power analysis employing GPower (*N* = 109, α = 0.05, two-tailed, power = 80%) revealed that the sample was sufficiently powered to detect a minimum effect size of *f*^2^ = 0.07.

### 2.2. Procedure

Upon arrival, the participants read a scenario (see Appendix A) that described the story of a student, Robert, who neglected his duty on a team project (i.e., in-class team presentation), resulting in poor performance and grades for both himself and his teammate, Chris. The scenario was created by combining two existing scenarios, one from de Hooge et al. [24] for the shame event, and the other from de Hooge, Nelissen, Breugelmans, and Zeelenberg [25] for the guilt event, so that the participants could interpret the described event as either a guilt- or shame-inducing experience for the protagonist. Specifically, poor performance (e.g., stumbling) in front of peers typically elicits shame, and the appraisal that the protagonist is responsible for the poor team performance and the bad grade of his teammate evokes feelings of guilt [26]. To manipulate the degree of future opportunity, the information was varied, as the team either had one more presentation (for the high future opportunity condition) or no more (for the low future opportunity condition) presentations in the course. Then, all the participants read a story about Robert at home, reflecting on the day’s events in class and choosing an avatar to express the way he felt at present on his Internet blog. The participants viewed the image on a computer screen. Afterwards, they indicated their beliefs about the intensity of the guilt/shame experienced by Robert, using a single item for each emotion (i.e., “*How [ashamed/much guilt] do you believe Robert would feel?*”; 1 = *no shame/guilt at all*, 7 = *a great deal of shame/guilt*). Participants then rated their expectations about future change of the target person (i.e., “*To what extent do you believe that Robert would try to change his attitude toward, and behavior during, teamwork in the future?*”; 1 = *not at all*, 7 = *very much*) At the end, the participants completed the 12-item (e.g., “My convenience is a big factor in the decisions I make or the actions I take”) CFC scale [19].

## 3. Results

### 3.1. Perceptions of Guilt and Shame

**Figure 1:**
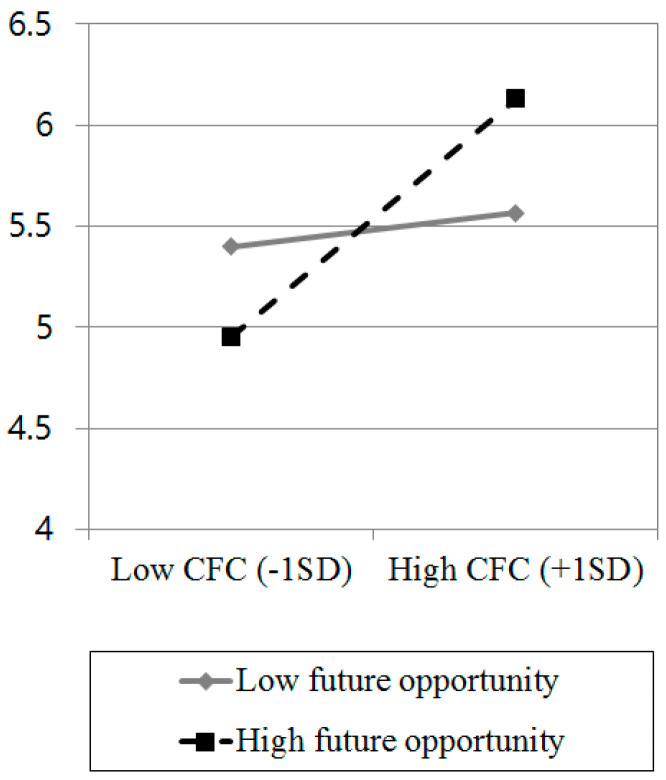
Guilt intensity rating as a function of consideration of future consequences (CFC) (high vs. low) × future opportunity (high vs. low) interaction.

Across conditions, the participants rated the target as experiencing guilt (*M* = 5.52, *SD* = 1.08) and shame (*M* = 5.51, *SD* = 1.16). A multiple regression analysis on guilt rating was conducted to test the hypothesis that the participants in the high future opportunity condition would perceive more intense guilt on the target’s face than those in the low future opportunity condition, but only if they were high in consideration of future consequences. The guilt intensity ratings were regressed on future opportunity (1 = low, 2 = high), CFC score (mean-centered), and the future opportunity × CFC interaction term. Supporting the hypothesis, the results showed that only the interaction between future opportunity and CFC significantly predicted the perceived intensity of guilt, *b* = 0.07, *SE* = 0.03, *t*(99) = 2.22, *p* = 0.029, total *R*^2^ = 0.17 (see Figure 1). Further simple slope analyses within the interaction [27] showed that the participants with high CFC (1 *SD* above the mean) viewed the target as experiencing greater guilt when the target had high rather than low future opportunity, *b* = −0.56, *SE* = 0.27, *t*(99) = −2.04, *p* = 0.044, 95% CI [−1.10, −0.02], whereas people with low CFC (1 *SD* below the mean) did not show any differences in their perceptions of guilt in between high and low future opportunity conditions, *b* = 0.45, *SE* = 0.27, *t*(99) = 1.64, *p* = 0.104, 95% CI [−0.09, 0.99]. For the shame intensity rating, we conducted the same regression analysis and found that there were no significant predictors, *p*s > 0.096.

### 3.2. Change Expectation

A similar analysis was conducted using the change in expectation rating as a dependent variable. Consistent with the findings in the guilt intensity rating, only a significant interaction effect emerged, *b* = 0.08, *SE* = 0.03, *t*(99) = 2.44, *p* = 0.017, 95% CI [0.02, 0.15], total *R*^2^ = 0.14, indicating that people with high CFC expected greater future change when the target had high rather than low future opportunity, *b* = 0.70, *SE* = 0.31, *t*(99) = 2.29, *p* = 0.025, 95% CI [0.09, 1.30], whereas people with low CFC showed no significant difference between high and low future opportunity conditions, *p* = 0.246.

### 3.3. Mediation Analysis

**Figure 2:**
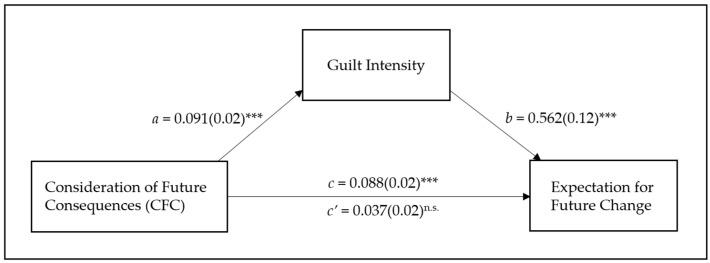
Mediational analysis for the high future opportunity conditions: coefficients (Standard errors) for the *a* pathway, *b* pathway, and *c* pathway. ***: *p* < 0.001.

To further investigate whether the guilt intensity ratings meditated the effect of CFC on expectations of change, especially when the future opportunity was high, a bootstrapping-moderated mediation analysis with 5000 iterations [28] was conducted. In the analysis model, future opportunity served as a moderator, and the guilt rating served as a mediator. The results revealed a significant conditional indirect effect of CFC on the change expectation through the perceived intensity of guilt, with a 95% CI [0.02, 0.10]. To examine the specific hypothesis about the conditional indirect effect, two separate bootstrapping mediation analyses [29] were conducted: one for the high future opportunity and another for the low future opportunity condition. The results showed that the 95% confidence interval for the indirect path coefficient excluded zero in the high, CI [0.02, 0.10], but not the low, CI [−0.01, 0.04], future opportunity conditions, suggesting that the CFC scores indirectly influenced the future change expectation through the perceived intensity of guilt when there was a high future opportunity (see Figure 2). Thus, the people who cared more about distant (vs. immediate) future consequences were more likely to perceive guilty feelings on the target’s face, and, in turn, expected that the target person would change his behavior to a greater degree.

## 4. Discussion

The present study examined whether uninvolved social observers infer greater future change from a target person’s guilt expressions compared to the target’s shame expressions, and whether such differences lead to emotion perceptions. Supporting the hypothesis, the results showed that the participants who cared about future consequences (i.e., high CFC) viewed the target person as experiencing more intense guilt when they believed that the target person would have a future opportunity (vs. no future opportunity) to change the outcome of the event. The participants with low CFC did not differ in their guilt ratings between future-opportunity conditions. On the contrary, shame intensity ratings did not differ across conditions. Thus, the present findings suggest that perception of others’ feelings of guilt and shame can be guided by how much opportunity exists for the target person to fix past misdeeds and how much the observers care about the implications for future change.

Importantly, the participants’ ratings of expectation of change showed a similar interaction pattern as their guilt ratings. It should also be noted that the guilt intensity rating fully mediated the effect of CFC on the ratings of expectation for future change when the future opportunity was high, but not when it was low. The present findings indicate that people expected greater future change from the target’s expression of guilt compared to shame.

The present study sought to differentiate between shame and guilt in the eyes of the observers. This study demonstrated that when the implications of self-conscious emotions for future change become important to the observers (i.e., when future opportunity is high and when it matters to the observers), participants tend to view guilt as more intense, but not shame. Thus, the present study suggests that people believe that guilt, rather than shame, signals greater potential for future change, and that this subtle difference in people’s theory of mind is reflected in the functional perception of others’ self-conscious emotions. The findings of this study suggest that, when an individual feels guilty, one should try to explicitly express guilt in public, as it will form a positive expectation of behavioral change in the future. Consequently, this favorable environment (i.e., that framed by social expectations) would facilitate corrective or desirable behavior [30]. This would be even more pronounced when observers are concerned about future consequences.

The present study contributes to the literature on emotions by providing an elaborate context (i.e., highly future-relevant situation) that helps social observers effectively differentiate between the social meanings of guilt and shame. Additionally, the present study has implications for a broader population. For example, research on children with autism spectrum disorder (ASD) has shown that children with ASD are less able to recognize self-conscious emotions [31,32]. Exploring situational contexts, such as future opportunities that may help children with ASD to better recognize and interpret self-conscious emotions, would be an important line of research. Furthermore, this study advances the literature on individual differences in the consideration of future consequences. By systematically varying the event repeatability to manipulate the future opportunity, the present study introduces a paradigm that researchers can employ to investigate any effect of individual differences in the consideration of future consequences.

The present study has several limitations. First, this study’s findings were limited to explaining the psychology of uninvolved observers. This finding may not be generalizable when a perceiver is involved in a moral situation (e.g., an injured party). For example, Giner-Sorolla, Kamau, and Castano [33] found that shame, but not guilt, expressed by a perpetrator reduces insult feelings among apology recipients. Presumably, for the involved perceivers, the social meaning of the perpetrator’s self-conscious emotions would be highly complicated by many factors, including group membership, motivation for retribution, and the previous interaction history. Second, the majority of the participants were white Americans, representing the perspective of individualistic culture. Given that sociocultural contexts can shape the self (see [34,35] for a review) and that emotion is regulated by cultural norms and values (e.g., [36]), the social meanings of self-conscious emotions can be subject to cultural variations (see [37,38] for a review). For example, in cultures that value shame, the motivation to change one’s behavior is more closely linked to shame than to guilt [39].

## 5. Conclusions

To conclude, shame and guilt, highly interchangeable self-conscious emotions, can be distinguished in the eyes of an observer, provided that situational contexts such as future opportunity and individual differences in sensitivity to future matters exist.

## Figures and Tables

**Figure A1:**
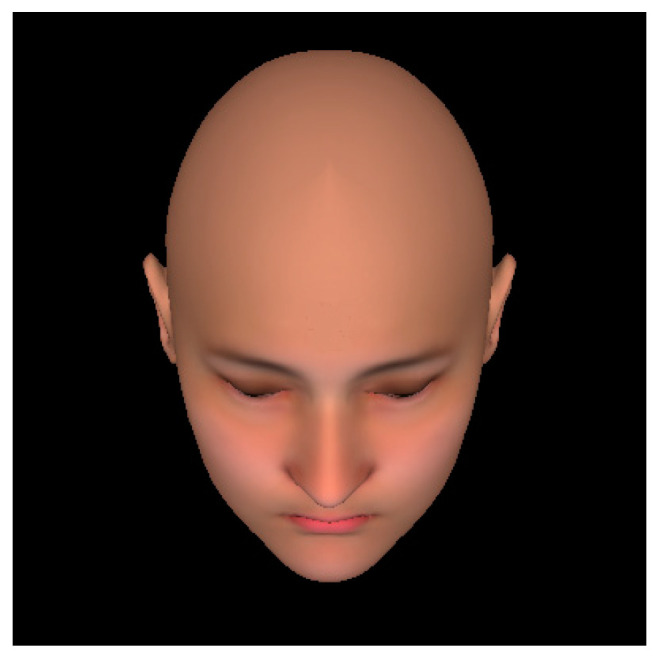
Image generated by FaceGen Modeller 3.5.

## Data Availability

The data presented in this study are available from the corresponding author upon request.

## Appendix A

Robert and Chris are taking a class in which every student has to give a team presentation [two team presentations]. An exam was given in the class last week, and Robert received the highest score in the class. Robert and Chris are now working on the first team presentation together. Because Robert just received the best score on the exam, and because he does not enjoy giving presentations, Robert hardly puts any effort into preparing the presentation. As a consequence, Chris winds up doing most of the work. When it comes time to do the presentation in front of the class, Robert’s part of the presentation goes very poorly. He stumbles over his own words, his arguments are weak, and by the end of the presentation it is clear that no one in the class understood what he was talking about. Moreover, when members of the class ask him questions, his answers are vague and suggest that he has little mastery of the subject. A short time later, Robert and Chris receive a low grade for the presentation because of Robert’s poor performance.

Robert and Chris will not be giving any more presentations to the class. This was their one opportunity to give a presentation [Two weeks from now, Robert and Chris will be giving one more presentation to the class].

When Robert goes back to his dorm room that day, he reflects on his experience in the class and blogs about his thoughts. He chooses to use an avatar to express the way he is feeling at the moment.
